# Notes and News

**Published:** 1903-04-25

**Authors:** 


### Notes and News.

On Sunday, June 7th, their Majesties the King and Queen will attend the afternoon service at St. Paul's Cathedral in connection with the Hospital Sunday Fund.

On June 8th her Royal Highness the Princess of Wales will open the new nurses' home in connection with the British Lying-in Hospital.

A concert will be held at the Queen's Hall on May 28th, under the patronage of the Princess of Wales in aid of the Children's Hospital, Paddington Green.

On May 4th the Duke of Cambridge will attend a dinner at the Mansion House in aid of the London Hospital.

The annual dinner of the Royal Hospital for Incurables, Putney, will take place on May 15th at the Grand Hotel, Charing Cross. Sir F. Dixon-Hartland will preside.

By the death of Prebendary Kitto the London Hospitals have lost an old friend. During his first incumbency he became honorary secretary of the Poplar Hospital, later he was associated with the London Hospital, and afterwards with Charing Cross Hospital. He was one of the original members of the council of the Hospital Sunday Fund brought together by Sir Sidney Waterlow.

"The Great Physician" is the title of a fine painting by Gabriel von Max, the great German painter, which has been magnificently reproduced as a gravure. The picture represents Christ the Physician sitting by the couch of the dead daughter of Jairus. The subject is treated with sympathy, grace, and breadth, and the rendering of the young girl is simple and poetic. The picture breathes solemnity and pathos, and inspires the growing admiration of the beholder. It is a most appropriate work for the adornment of the walls of institutions devoted to the care of the sick, the motive being both hopeful and religious. The publishers of the beautiful gravure are Messrs. Nicolaus Lehmann, of Prague.

The late Mr. George Braithwaite Lloyd, of Birmingham, has bequeathed £250 each to the General Hospital and the General Dispensary, and £100 each to the Queen's Hospital, the Birmingham and Midland Hospital for Women and Children, the Homœopathic Hospital, the Eye Hospital, and the Birmingham Lying-in Charity.

Bequests to hospitals are not so numerous that it is a small matter when one of £1,200 miscarries simply through want of a little care on the part of the signatories of the will. Yet such has been the misfortune recently of the Chelsea Hospital for Women in the matter of the late Mr. William Toby's estate. The testator signed the will in his bedroom, the witnesses, unfortunately, took it into another room before signing it, with the result that a worthy institution is deprived of a useful contribution.

Alderman Treloar informs us that, after paying for the Christmas dinners in the Guildhall, defraying the cost of 6,077 hampers, and the expenses of distribution, he is left with a balance in hand of £208 10s. 9d. He proposes to utilise this sum to assist in well-authenticated instances of need, and provide these invaluable reliefs to bodily suffering, and if the £208 permits he may perhaps be able to supplement this help by the gift of a small sum to the most deserving, whereby a breath of fresh air may be obtained in the country.

A testimonial, together with a purse, has been presented to Mr. Henry James Tresidder, upon his retirement from the post of secretary to the National Orthopædic Hospital, signed by members of the committee and medical staff, in recognition of the valuable services rendered by him during the period from August 1893 to November 1902. Mr. Tresidder also received letters from the President, Treasurer, and the Ladies' Committee with kind expressions and good wishes, and the matron and nurses presented a travelling bag "in grateful remembrance of many kindnesses and much consideration." Mr. Tresidder called the first meeting of the Surgical Aid Society, and read the paper forming the basis for its formation, and was elected secretary, from which position he had to retire owing to other responsibilities.

The Didsworthy Sanatorium at South Brent for the treatment of phthisical patients has just been opened. The building is a mansion accommodating 14 patients, and standing in an estate of 67 acres, to the south of Dartmoor, and the total cost of its purchase and equipment has been £6,500, raised by public subscription, the Earl of Mount Edgcumbe being the chairman of the committee. It is calculated that each bed will cost £75 per annum, and will accommodate three or four patients. Devonport and Plymouth Boards of Guardians are each supporting a bed, and Plymouth Corporation have resolved to retain two. Interest in the open-air treatment is increasing in the West. The Devon and Cornwall County Councils have had the matter before them, and should Didsworthy prove the success its promoters anticipate it will doubtless give an impetus to the formation of county institutions.

